# Infra Red Spectroscopy of the Regulated Asbestos Amphiboles

**DOI:** 10.3390/min8090413

**Published:** 2018-09-18

**Authors:** Giancarlo Della Ventura, Ruggero Vigliaturo, Reto Gieré, Simone Pollastri, Alessandro F. Gualtieri, Gianluca Iezzi

**Affiliations:** 1 Dipartimento di Scienze, Universita Roma Tre, Largo S. Leonardo Murialdo 1, I-00146 Rome, Italy; 2 Istituto Nazionale di Fisica Nucleare, Via E. Fermi 40, Frascati, I-00044 Rome, Italy; 3 Department of Earth and Environmental Science, University of Pennsylvania 240 S. 33rd Street, Hayden Hall, Philadelphia, PA 19104-6316, USA; ruggero.vigliaturo@gmail.com (R.V.); giere@sas.upenn.edu (R.G.); 4 Center of Excellence in Environmental Toxicology, University of Pennsylvania, Philadelphia, PA 19104, USA; 5 CERIC-ERIC, ss 14, Km 163.5, Basovizza, I-34149 Trieste, Italy; simone.pollastri@ceric-eric.eu; 6 Department of Chemical and Geological Sciences, University of Modena and Reggio Emilia, Via Campi 103, Modena, I-41125 Italy; alessandro.gualtieri@unimore.it; 7 Dipartimento di Ingegneria & Geologia (INGEO), Università G. d’Annunzio, Via Dei Vestini 30, I-66013 Chieti, Italy; g.iezzi@unich.it

**Keywords:** regulated asbestos amphiboles, FTIR spectroscopy, FEG-FESEM, TEM, SAED patterns

## Abstract

Vibrational spectroscopies (Fourier Transform Infra Red, FTIR, and Raman) are exceptionally valuable tools for the identification and crystal-chemical study of fibrous minerals, and asbestos amphiboles in particular. Raman spectroscopy has been widely applied in toxicological studies and thus a large corpus of reference data on regulated species is found in the literature. However, FTIR spectroscopy has been mostly used in crystal-chemical studies and very few data are found on asbestos amphiboles. This paper is intended to fill this gap. We report new FTIR data collected on a suite of well-characterized samples of the five regulated amphibole species: anthophyllite, amosite, and crocidolite, provided by the Union for International Cancer Control (UICC) Organization, and tremolite and actinolite, from two well-known occurrences. The data from these reference samples have been augmented by results from additional specimens to clarify some aspects of their spectroscopic features. We show that the FTIR spectra in both the OH-stretching region and in the lattice modes region can be effective for rapid identification of the asbestos type.

## 1. Introduction

The term “asbestos” is a misleading non-scientific commercial term [1], which commonly refers to silicate minerals with fibrous morphology [2]. From a legal perspective, six fibrous minerals are regulated as asbestos minerals: five species of the amphibole group, and chrysotile, which belongs to the serpentine group of the layer silicates [3–5]. From a mineralogical point of view, chrysotile has only limited variations from its ideal crystal–chemical composition described as Mg_3_Si_2_O_5_(OH)_4_ [6], whereas amphiboles exhibit extremely wide ranges in composition, even in samples from the same outcrop, in the same rock, or within the same (zoned) crystal [7–10]. Such chemical variability results in significant challenges for identification and classification of amphiboles, implying difficulties for health and legal issues arising from exposure to the amphibole fibers [11,12]. Another critical aspect concerns the textural features describing “asbestiform,” “fibrous,” or “acicular” minerals [9,13–16]. According to the criteria of the World Health Organization (WHO), a regulated “fiber” is an elongated particles with a length ≥5 μm, a width ≤3 μm, and an aspect ratio (length:width) ≥3:1. Needle-like crystals not fulfilling this criterion are more correctly termed “acicular”; we will use these definitions throughout this text.

Accurate and full characterization of asbestos fibers and their host rocks requires a combination of several analytical techniques, most notably optical microscopy, scanning and transmission electron microscopy (SEM and TEM), X-ray diffraction (XRD), and vibrational spectroscopy (Fourier Transform Infra Red, FTIR, and Raman) [16]. Both FTIR and Raman spectroscopy have been applied for the identification of fibers in environmental or biological/medical applications, where the identification of the sample was the main goal. However, a careful examination of the literature reveals that, although Raman spectra are available as reference data for the correct fiber identification, reference FTIR data for the regulated mineral species are lacking.

To fill this gap, we provide here FTIR data for well-characterized samples of the five regulated amphibole species: anthophyllite, amosite, and crocidolite, provided by the Union for International Cancer Control (UICC) Organization, as well as tremolite and actinolite, which were from two well-known occurrences and were diffusively used in several previous studies. Where needed, the data from these reference samples have been augmented by, and compared with, results from additional specimens to clarify some of the spectroscopic features. All mineral samples used in this study have been examined by Field Emission Gun Environmental SEM (FEG-ESEM) and TEM prior to their characterization by FTIR. The FTIR data presented shall serve as a reference data set when dealing with fibrous materials in environmental or toxicological studies.

## 2. Vibrational Spectroscopy Applied to the Crystal Chemistry of Fibrous Amphiboles

FTIR and Raman spectroscopy are frequently used for the characterization of earth and analogue materials [17–19]. Both methods have been used extensively for rapid identification of asbestiform minerals and the characterization of their site occupancies [8,20–23]. Vibrational spectra can be collected either on powders or on single crystals, requiring only a very small amount of material (mg), such as tiny crystals with sizes ranging from some μm to few tens of μm. The spatial resolution of conventional Raman spectroscopy is better than that of FTIR spectroscopy, but similar to that of an IR beam at a synchrotron source [24,25]. Commonly, FTIR data are collected on few mg of powdered asbestos samples dispersed in a salt matrix (e.g., KBr, NaCl) to form a pellet (see below). Asbestos materials can also be analyzed by diffuse-reflectance IR spectroscopy (DRIFT), where the IR beam is scattered by the rough sample surface, collected by a mirror, and then sent to the detector [26]. This technique is helpful when studying cement-asbestos, because the powder to be analyzed can be easily scratched off a wall, a brick, or a roof tile by using an abrasive paper, and subsequently placed onto the DRIFT sample holder without any further preparation.

The vibrational spectrum of an amphibole (as well as of all hydrous silicates) is conventionally divided into two ranges: the higher wavenumber region (4000–3000 cm^−1^) where typical bands due to water/OH stretching are observed, and the lower wavenumber range (<1200 cm^−1^), where bands resulting from longer bonds, such as those of the tetrahedral skeleton, the cation-oxygen (≥six-fold) polyhedral, or the bending/libration modes, are found. The near-infrared (NIR) region, at wavenumbers 10,000–4000 cm^−1^, although rarely used, is also of significant interest due to the possible applications in remote sensing [27].

Amphiboles may crystallize in several different space groups, but the *C*2/*m* is by far the most common [28]. Their OH-stretching (3000 to 4000 cm^−1^) FTIR and Raman spectra are very similar and show identical band multiplicity and band wavenumbers [25,26,29]; by contrast, band intensities can be significantly different, a feature that has important consequences in quantitative analysis [30]. At low frequency, the lattice mode region (<1200 cm^−1^) can display a large number of peaks for a *C*2/*m* amphibole [29]. Such complex spectra, typically consisting of many overlapping bands, prevent a reliable assignment of the observed modes to a single molecular motion. Moreover, in this low-frequency region, IR and Raman spectra are significantly different [26]. For mineral identification and crystal-chemical studies, however, the spectral results can be used by comparison with a relatively large dataset available in the literature, collected on samples spanning the complete compositional space of environmental/biological interest. Here, we focus on completing this set of reference data for FTIR, which are lacking or scattered throughout the literature.

**Figure 1.**
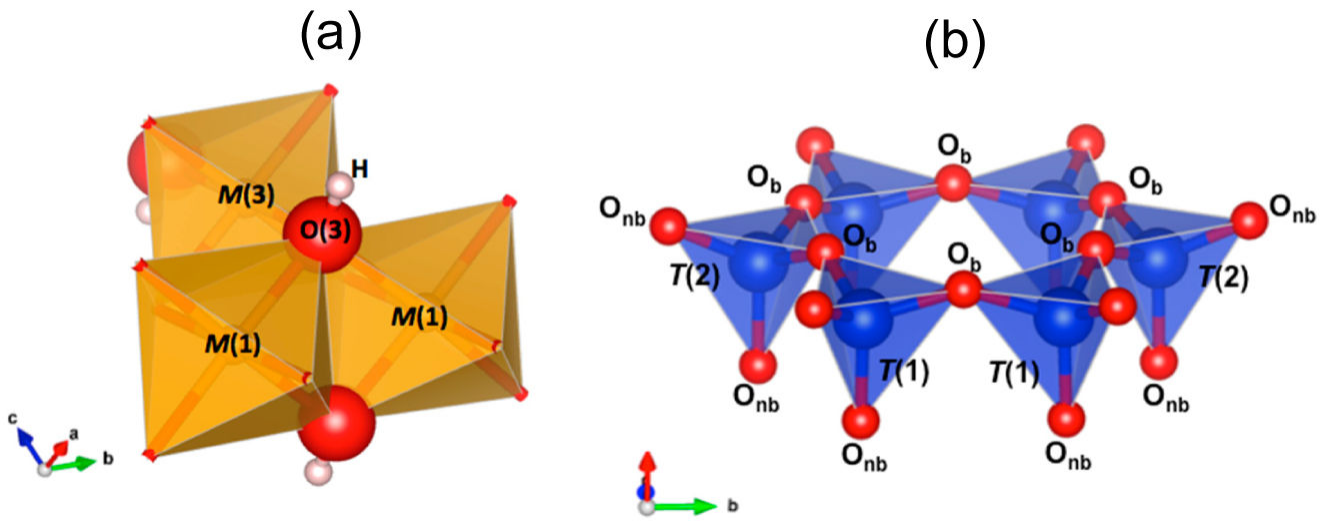
(**a**) Local octahedral configuration around the OH group in amphiboles. (**b**) Schematic representation of part of the double-chain of tetrahedra in amphiboles. O_b_ = bridging oxygen, O_nb_ = non bridging oxygen.

In amphiboles, the OH group is directly bonded to three cations in six-fold coordination, labelled *M*(1) end *M*(3), forming tine base of a nearly equilateral triangle around the OH group (Figure 1a). The O–H distance is extremely short (≈0.96 Å [31]), and consequently, the OH-stretching modes occur at very high wavenumbers (3750–3600 cm^−1^).

This frequency range is almost invariably free from interferences by other absorbing species, which represents a clear advantage of O–H stretching spectroscopy. The exact position (i.e., energy) of the principal OH-stretching band(s) scales with the relative strength of the O-H bond that is also dependant on the degree of hydrogen bonding between the H atom and the surrounding O atoms. In other words, both the strength and the length of the O–H bond are extremely sensitive to arty local arrangement of atoms around the OH group. This aspect makes IR and Raman spectroscopy very important tools for crystal-chemical studies of hydrous silicates. Details on how the exact position of the bands observed in the OH-stretching spectra of amphiboles can be used as a tool for their characterization can be found is a vast body of literature (see [21,32] for a complete list of references). Specifically, applications to the study of fibrous amphiboles can be found in References [8,26,33].

## 3. Materials and Methods

A list of the samples used for this investigation, including mineral formulae and information on their occurrence, is given in Table 1.

Powder FTIR spectra were collected at Roma Tre University using a Nicolet iS50 spectrometer, equipped with a Globar source, a KBr beam-splitter, and a DGTS (Deuterated Triglycine Sulfate) detector. Sixty-four spectra were averaged for both sample and background, for a nominal resolution of 4 cm^−1^. Samples were prepared as KBr pellets, using 5:150 and 0.5:150 mg ratios for the 3000–4000 and <1200 cm^−1^ regions, respectively. Single-crystal FTIR spectra in the OH-stretching medium-infrared (MIR) were collected with unpolarized light using a Bruker Hyperion 3000 microscope equipped with an MCT (Mercury Cadmium Telluride) detector and a KBr beam splitter at Istituto Nazionale di Fisica Nucleare (INFN, Frascati, Rome).

For the SEM analysis, the mineral powders were dispersed in 2-propanol and ground by hand, using an agate mortar for 3 min, resulting in a homogeneous suspension. An FEI Quanta 600 Field-Emission-Gun Mark II Environmental Scanning Electron Microscope (FEG-ESEM) was used to investigate the samples. Environmental SEM allows for observation of the sample without the need of a conductive coating, and thus, the 2-propanol suspensions containing the amphiboles were directly transferred onto a conductive carbon tape fixed onto a SEM stub (12.7 mm diameter). The instrument was set to environmental mode with a voltage of 15 kV and a chamber pressure of 0.53 Torr. Variable beam dimension was used to optimize imaging and semi-quantitative chemical analysis (in general, sizes 3 and 6, respectively). Chemical data obtained with the SEM through energy-dispersive X-ray (EDX) spectroscopy were used exclusively to confirm the identity of the studied specimen.

TEM analyses were conducted on a JEOL 2010F TEM and involved a combination of imaging and high-resolution (HRTEM) techniques together with selected area electron diffraction (SAED). The microscope was operated at 200 kV combining different sets of objective apertures, selected area apertures, and camera lengths to optimize the observations. All examinations were performed on a minimum of 50 fibers.

## 4. Results and Discussion

### 4.1. OH-Stretching Spectra and Crystal Chemistry

#### 4.1.1. UICC Anthophyllite Asbestos

Anthophyllite has the ideal end-member composition ^A^□^B^(Mg)_2_^C^(Mg)_5_^T^Si_8_O_22_^W^(OH)_2_. According to the most recent IMA (International Mineralogical Association) classification, anthophyllite belongs to the magnesium-iron-manganese subgroup, defined by ^B^(Ca + ΣM^2+^)/ΣB ≥ 0.75, BΣM2+/ΣB > BCa/ΣB. Amphiboles of this subgroup may be orthorhombic (space groups *Pnma* or *Pnmn*) or monoclinic (space groups *C*2/*m* or *P*2_1_/*m*). End-member anthophyllite is orthorhombic, with a potential solid-solution toward ferro-anthophyllite ^A^(□)^B^(Fe^2+^)_2_^C^(Fe^2+^)_5_^T^(Si)_8_O_22_^W^(OH)_2_. The monoclinic *P*2_1_/*m* and Mg-rich polymorph of the series is named cummingtonite, where a low amount of Fe^2+^ at both B- and C-sites stabilizes the *C2/m* polytype [42].

Anthophyllite has a color ranging between dull green to white; it typically occurs in metamorphic rocks of the lower to middle amphibolite facies and in serpentinized ultrabasic rocks [7], where it almost invariably grows as distinctly fibrous crystals. Anthophyllite was industrially employed in limited amounts, mainly for insulation products and construction materials; it frequently occurs as a contaminant in chrysotile asbestos, vermiculite, and talc [7]. The main commercially exploited deposits are those in metamorphosed dunites and harzburgites of Eastern Finland (Paakkila and Maljasalmi), where anthophyllite was formed as a replacement of olivine and orthopyroxene during the serpentinization of the host rock. The UICC sample investigated here was from Paakkila (Table 1). Similar occurrences are known in the U.S., such as in Idaho, Georgia, and North Carolina [43].

According to the X-ray powder diffraction (XRPD) analysis given in References [34,35], the studied sample consisted of amphibole with impurities of biotite, clinochlore/vermiculite, and talc. The refined unit-cell data (Table 2) were in agreement with previous data for similar amphiboles [28].

**Figure 2.**
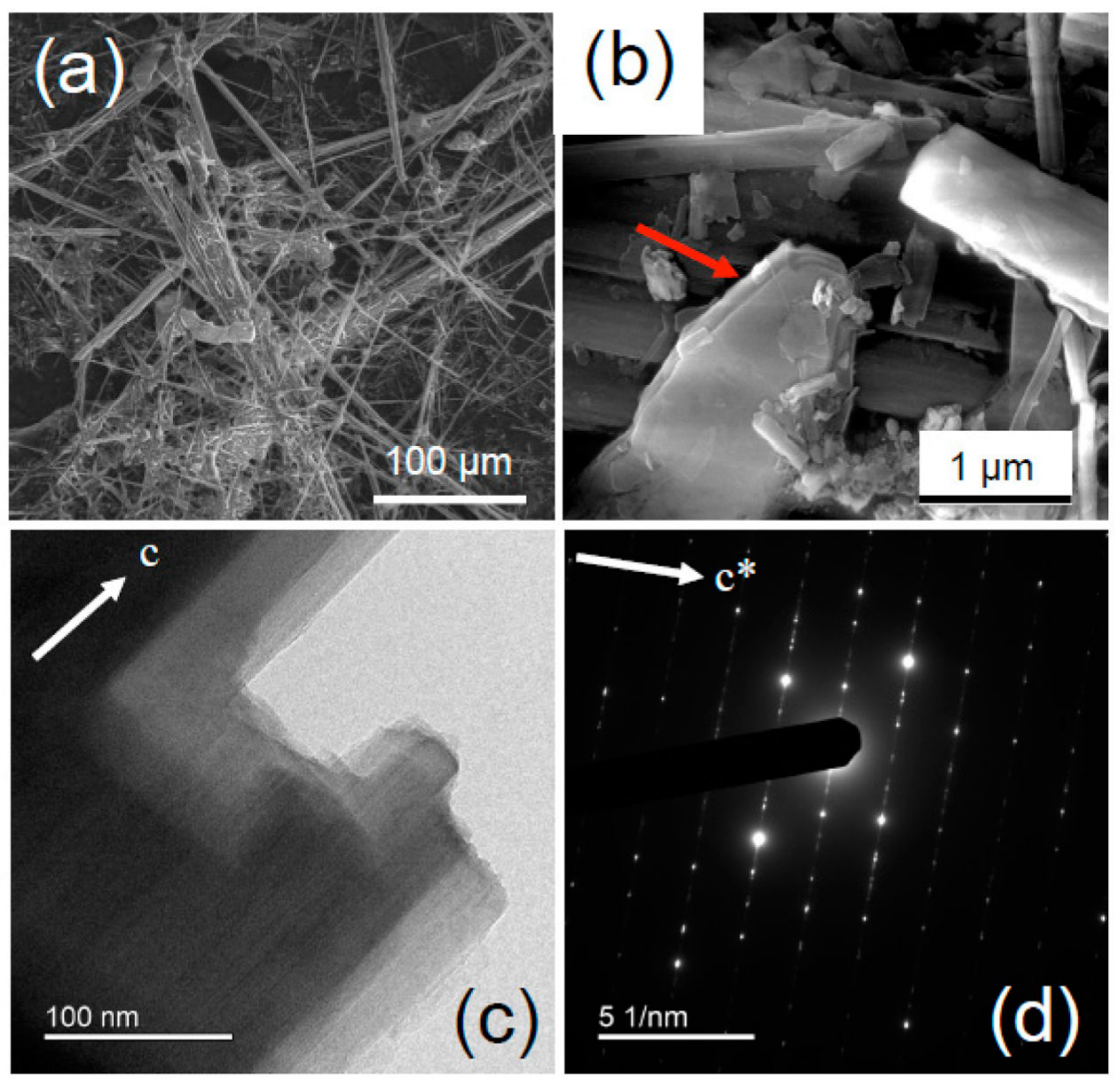
UICC standard anthophyllite Finnish NB #4173-111-5 sample (Table 1). Representative FEG-ESEM images at relatively low (**a**) and high (**b**) magnification, where a platy talc particle (red arrow) is visible. (**c**) High-resolution TEM image highlighting edge features of the fiber viewed along the c axis, and (**d**) SAED pattern parallel to the c* axis.

FEG-ESEM images (Figure 2a) show that the sample consisted of asbestiform to acicular crystals with dimensions averaging 1 μm in width and more than 100 μm in length. Larger bundles of acicular crystals, a few tens of μm in width, were also common. In agreement with the XRPD data, lamellar and platy particles of talc were recognizable in the FEG-FESEM images (Figure 2b). The fibers showed very straight edges and low amounts of amorphous material along the surfaces. Several stepped features could be detected at the apex and along the edges of the fibers (Figure 2c).

The SAED patterns obtained by TEM display the typical amphibole streaking normal to the c axis (Figure 2d), which resulted from diffuse chain-width errors (chain multiplicity faults: CMF). Published microchemical data (Table 3) show that the UICC anthophyllite contained appreciable amounts of iron; Mössbauer and XAS (X-ray Absorption Spectroscopy) data indicate that Fe was present entirely as Fe^2+^ [38]. Additional components include Mn (0.04 apfu) and trace amounts of Ni, Cr, and Ca.

**Figure 3.**
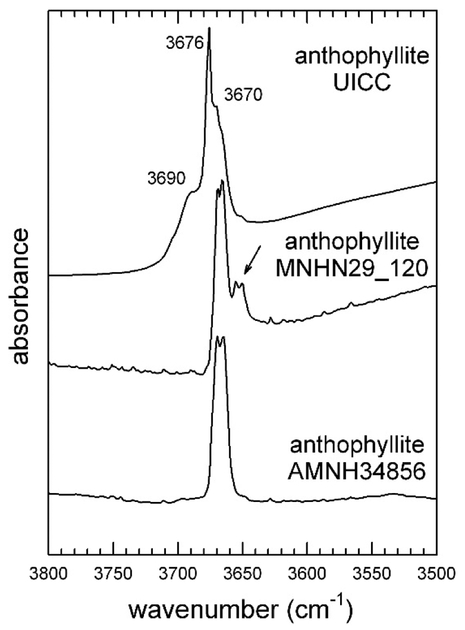
FTIR OH-stretching spectra of the studied UICC standard anthophyllite Finnish NB #4173-111-5 compared to sample MNHN29_120 and anthophyllite AMNH34856.

The FTIR spectrum in the OH-stretching region (Figure 3) of the UICC sample displays a relatively broad absorption consisting of several overlapping bands at 3690, 3676, and 3670 cm^−1^, with the 3676 cm^−1^ band being the sharpest and most intense. This type of pattern does not match the pattern expected by considering the local configuration of the OH group in amphiboles [21] with anthophyllite composition [45]; FTIR spectra of four (Mg, Fe^2+^) anthophyllites published in Reference [46] indeed show, in the OH-stretching region, four components, namely at 3666, 3654, 3639, and 3618 cm^−1^, whose relative intensities are correlated with the Mg/Fe^2+^ ratio at *M*(1,3). Ishida and Hawthorne [47] later reported that anthophyllite shows considerable fine structure in the form of band splitting of the principal OH-stretching region, indicative of an orthorhombic symmetry with two very similar but crystallographically independent OH groups [28].

To further corroborate the FTIR data, we collected infrared spectra of two well-characterized anthophyllite-group minerals for comparison (Table 1). For the amphibole from Norway (MNHN 29_102), we could collect a powder FTIR spectrum, whereas for that from Talcville (AMNH34856), we could collect only a single-crystal spectrum due to the scarcity of material. The anthophyllite from Norway shows (Figure 3) two absorptions, resulting from the presence of small amounts of Fe^2+^ at *M*(1,3) (Della Ventura, unpublished data), both showing fine structure, whereas the anthophyllite from Talcville shows only one doublet (Figure 3). This latter amphibole indeed represents a reference point for this species, because it exhibits nearly end-member composition; its complete crystal-chemical characterization [36], which included EMPA (Electron Micro Probe Analysis) and XRPD data, returned the formula ^A^Na_0.01_^B^(Mg_1.30_Mn_0.57_Ca_0.09_Na_0.04_)^C^(Mg_4.95_Fe_0.02_Al_0.03_)^T^(Si_8.00_)O_22_^W^(OH)_2_, with significant Mn at *M*(4) only,. Therefore, only Mg was present at the OH-coordinated *M*(1,3)-sites, besides trace amounts of Fe^2+^ and Al. In agreement with these results, only one split absorption was present in the OH-stretching region. The splitting factor was Δ = 5 cm^−1^, and was observed only at high resolution (≤2 cm^−1^), both in powder and single-crystal spectra. The observed splitting was thus direct evidence for the symmetry of the amphibole to be either *Pnma* or P*2*_*1*_*/m* Based on the above discussion, in the FTIR spectrum of the UICC anthophyllite, the sharp peak at 3676 cm^−1^ was assigned to talc, whereas the broad absorption at 3690 cm^−1^ could be assigned to clinochlore/vermiculite impurities [45]. According to the Rietveld refinement results of Reference [35], the UICC anthophyllite is orthorhombic *Pnma*, but the band splitting in the OH spectrum is not observable due to the overlap with the signals from the talc and clinochlore impurities. We also note that, although the sample contained significant Fe (Table 3), no absorptions due to local configurations, such as MgMgFe or MgFeFe, were observed in the spectrum, This is consistent with the fact that in the UICC anthophyllite, all ferrous iron was ordered at *M*(4), whereas tha *M*(1,2,3) octahedra had a composition of Mg = 5.0 apfu (atoms per fomula unit [35]. Anthophyllite MNHN 29_102 contained 13.0 wt% Fe, and the FTIR spectrum showed two split O-H absorptions (Figure 3), again consistent with a *Pnma* or *P*2_1_/*m* symmetry; however, the presence of the 3655–3650 cm^−1^ doublet (highlighted by the arrow in Figure 3) indicates that Fe^2+^ was partially disordered at *M*(1,3) in this sample.

#### 4.1.2. UICC Amosite (Grunerite Asbestos)

The commercial material “amosite”, an acronym for “Asbestos Mines of South Africa”, is commonly referred to as “brown asbestos”. It corresponds to the amphibole-group mineral grunerite, which is characterized by the ideal formula ^A^(□)^B^(Fe^2+^)_2_^C^(Fe^2+^)_5_^T^(Si)_8_O_22_^W^(OH)_2_ and monoclinic *C*2/*m* symmetry; the orthorhombic polymorph is instead labelled ferro-anthophyllite. A complete Fe^2+^-Mg solid-solution may exist along the monoclinic grunerite-cummingtonite series, which is correlated to a symmetry adjustment from *P*2_1_/*m* to *C*2/*m* [48,49]. Hence, and in spite of, the chemical simplicity involving the homovalent Mg-Fe^2+^ exchange, the amphiboles of the magnesium-iron-manganese subgroup can crystallize with different structures, a feature that is strongly correlated to the size of the B-site cations [50–53]. Notably, the Mg-end member anthophyllite is orthorhombic, the Mg-rich (or Fe-poor) cummingtonite is *P*2_1_/*m*, whereas the Fe-rich (or Mg-poor) grunerite is *C*2/*m* 654,55].

**Figure 4.**
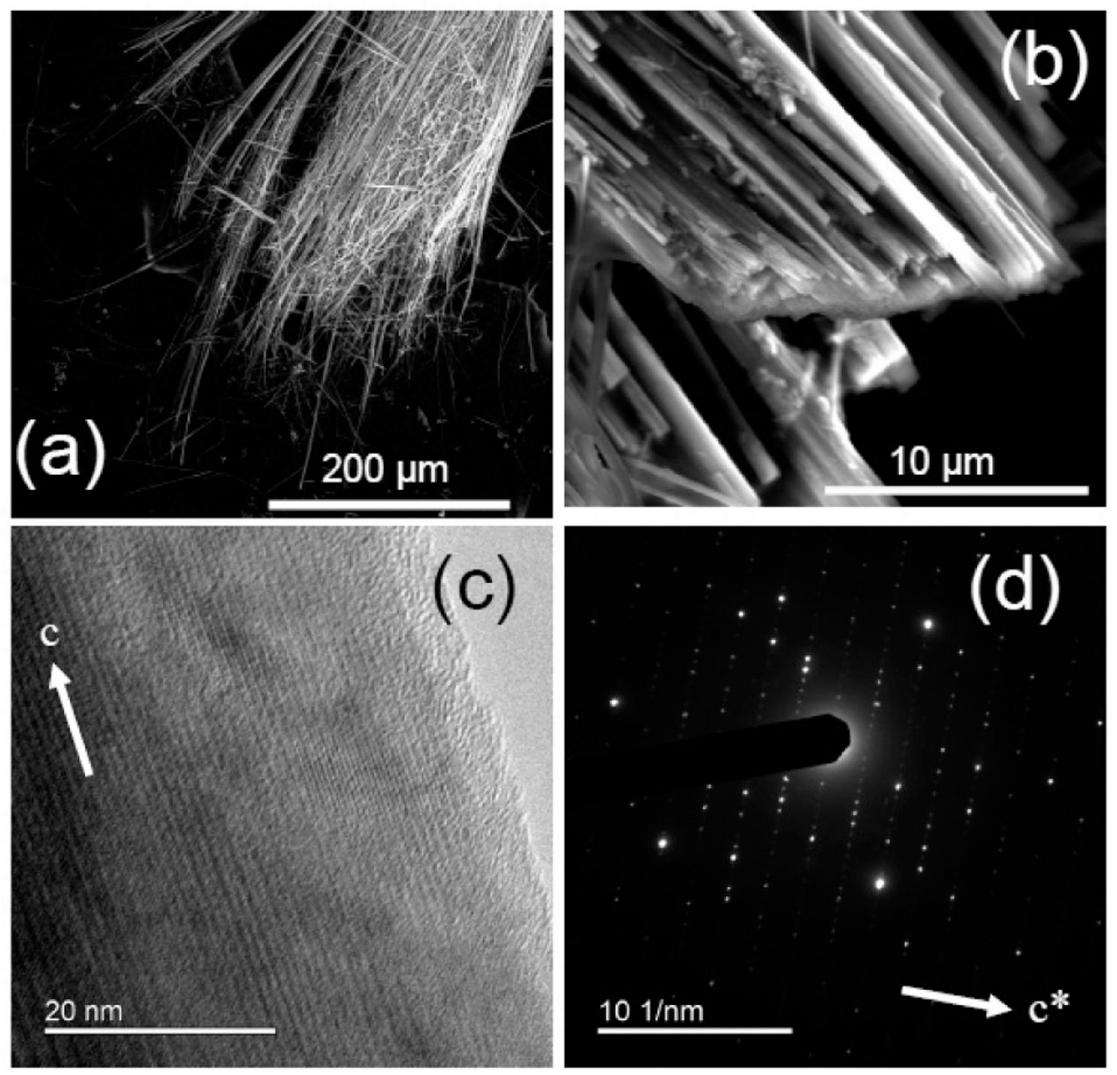
UICC amosite from South Africa NB #4173-111-4 (Table 1). Representative FEG-ESEM images at relatively low (**a**) and high (**b**) magnifications. (**c**) HR-TEM image documenting a limited-extent amorphous cover layer, and (**d**) SAED pattern parallel to tire c* axis.

**Figure 5.**
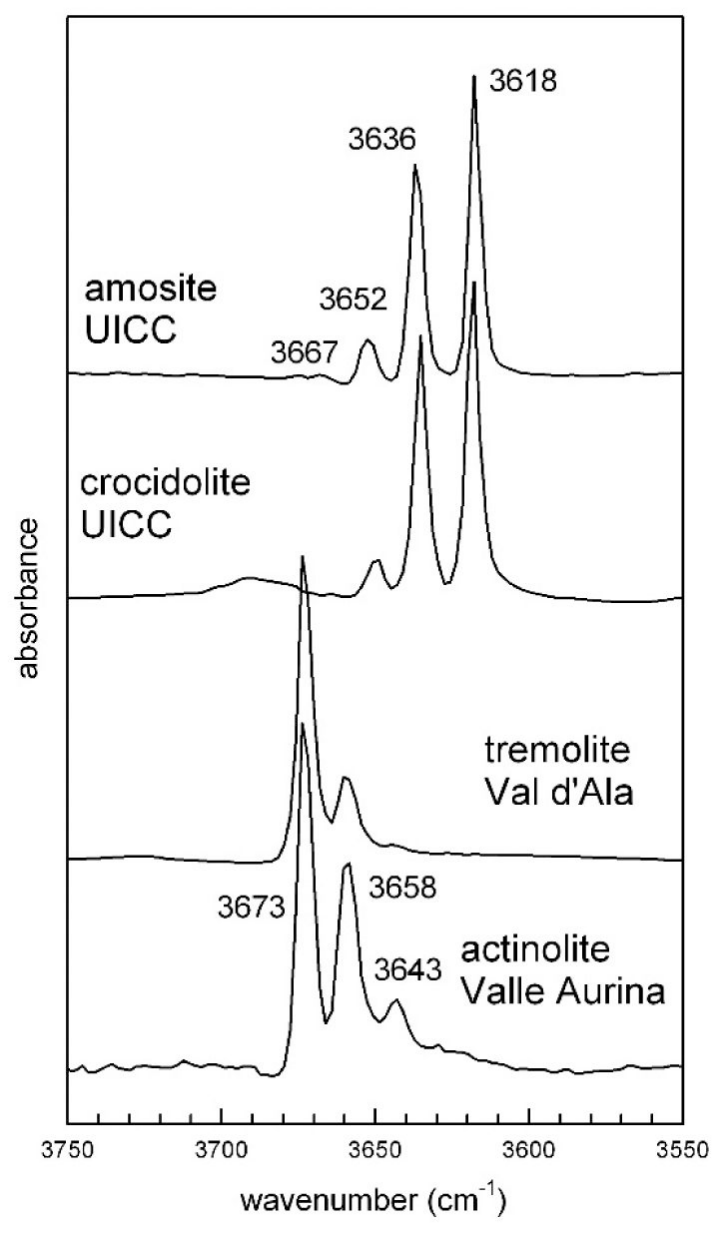
FTIR OH-stretching spectra of the studied amphiboles (Table 1): UICC amosite from South African NB #4173-111-4, UICC crocidolite; NB#4173-111-3, tremolite from Veil d’Ala, and actinolite from Valle Aurina.

The members of the cummingtonite-grunerite series are typically found in Ca-poor regionally and contact metamorphosed rocks; they are well known from metamorphosed banded iron formations (BIF). Amosite fibers, up to several cm long, occur interbedded within ironstones [56]. Mg-rich compositions are also reported from igneous environments [7]. Amosite was used most frequently in cement sheets and pipe insulation, insulating boards, ceiling tiles, and thermal insulation products. Although cummingtonite-grunerite amphiboles are relatively common, there is only one locality were these materials have been commercially exploited, i.e., the metamorphic iron formations of Eastern Transvaal (South Africa). We characterize here the standard UICC material, sourced from the Transvaal locality (Table 1). The same material was investigated with XRPD in Reference [35], showing it to consist of amphibole with impurities of calcite, hematite, and quartz. Rietveld refinement demonstrated that the sample is monoclinic *C*2/*m*, with the lattice parameters listed in Table 2, in agreement with previous data for similar amphiboles [28]. Specifically, the measured small *β* angle agrees well with monoclinic amphiboles hosting small divalent cations (Mg and/or Fe^2+^) at the B-site. FEG-FESEM images of the studied UICC amosite (Figure 4a,b) revealed that the sample consisted of ≈1 μm-wide fibres, which were arranged in parallel fashion to form elongated bundles with lengths of several hundred μm. In addition, the amosite fibers exhibited crystal defects (CMF), which were visible in HRTEM images (Figure 4c) and confirmed by streaking normal to the c* direction, as observed in the SAED patterns (Figure 4d). The SAED patterns display defined spots as well as satellite reflections (due to the presence of more than one crystallite in the selected area), which exhibit less intense streaking than those in anthophyllite. Many fibers have an extended amorphous surface cover, with a thickness ranging between 1 and 5 nm (Figure 4c). Inspection of the available chemical data for the UICC amosite revealed that this sample is close to the Fe-end member composition, but contained significant amounts of Mg (6.23 wt% of MgO) and ferric iron (3.9 wt% of Fe_2_O_3_). Trace elements included Ti, Cr, Ni, Mn, Ca, and alkalis (Table 3). The FTIR spectrum of the UICC amosite in the OH-stretching region (Figure 5) shows three well-resolved peaks at 3652, 3636, and 3618 cm^−1^, as well as an additional, very weak peak at 3667 cm^−1^. The spectrum is consistent with the distribution of Mg and Fe^2+^ at the C-sites, the observed bands being assigned to hydroxyl groups bonded to MgMgMg, MgMgFe^2+^, MgFe^2+^Fe^2+^, and Fe^2+^Fe^2+^Fe^2+^ octahedral clusters, from higher to lower frequency, respectively. Moreover, no bands due to the presence of Fe^3+^ are observed in this spectrum [57,58], demonstrating it to be fully ordered at the *M*(2)-site. The peaks of UICC amosite are sharp, with a nearly Lorentzian shape and without any hyperfine splitting such as that observed for anthophyllite (Figure 3). These features are indicative of a well-ordered *C*2/*m* structure. It is known since the pioneering work in References [59,60] that the relative intensities (peak area) of the four components observed in the OH-spectrum of amphiboles is correlated to the Fe^2+^/Mg occupancy at the hydroxyl-coordinated *M*(1,3) sites. This feature has been validated later with the analysis of several sets of synthetic solid-solution series with varying and controlled composition at the octahedral strip [61–65]. Deconvolution of the OH-spectrum of UICC amosite was relatively straightforward due to the peak sharpness and lack of band overlapping; following the method described in Reference [26], a value of ^*M*(1,3)^Fe^2+^ = 2.40 apfu was obtained, in excellent agreement with the value derived from site-occupancy refinement via the Rietveld method from synchrotron-radiation XRD data [35].

#### 4.1.3. UICC Crocidolite (Riebeckite Asbestos)

Crocidolite, the so-called “blue asbestos,” is the most popular and commercially employed amphibole asbestos. Mineralogically, it corresponds to the end-member riebeckite, which has the nominal formula ^A^(□)^B^(Na)_2_^C^(Fe^2+^_3_,Fe^3+^_2_)^T^(Si)_8_O_22_^W^(OH)_2_ and is classified in the sodic amphibole subgroup defined by ^B^(Na + Li)/ΣB ≥ 0.75, ^B^Na/ΣB ≥ ^B^Li/ΣB [42]; mineral species in this group invariably display the monoclinic *C*2/*m* symmetry. The chemical composition of riebeckite can vary substantially, primarily due to two main substitutions at the C-sites, i.e., Fe^2+^ ⇔ Mg and Fe^3+^ ⇔ Al [58]. These amphiboles are typical constituents of acidic igneous rocks, such as granites and quartz syenites, as well as high-grade metamorphic rocks. The fibrous varieties are essentially found in meta-ironstones, where the blue asbestos occurs in columnar and slip-fiber veins, whose origin is connected with rock deformation coupled to intense activity of metamorphic/metasomatic fluids. This extensively exploited asbestos material was used massively for insulation of steam engines, but also for some spray-on coatings, pipe insulation, plastics, and cement products. Notable occurrences of crocidolite are those of Bolivia, Western Australia (Hamersley Iron province), and South Africa (Transvaal and Cape Province). Here, we consider the UICC standard from South Africa (Table 1).

**Figure 6.**
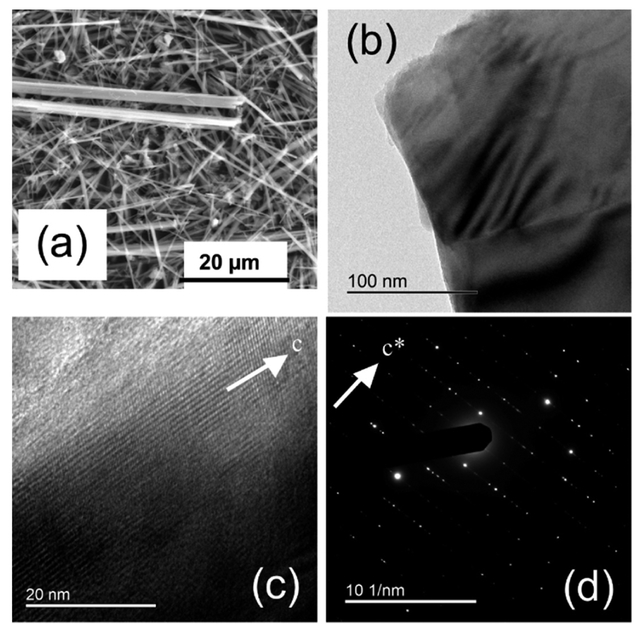
UICC croci dolite NB#4173-111-3 (Table 1). (**a**) Representative FEG-ESEM image of the typical texture of the sample at high magnification. (**b**) HRTEM image with Moiré fringes. (**c**) HRTEM image showing a transition region from a highly crystalline area (streaking along c is dearly visible) to a nearly amorphous boundary. (**d**) Representative SAED pattern parallel to the c* axis.

XRPD data for the amphibole studied here can be found in Reference [34]; accordingly, the sample consists of amphibole plus very minor amounts of hematite, magnetite, quartz, talc, and lizardite. The lattice dimensions of the amphibole are given in Table 2. A representative FEG-FESEM image (Figure 6a) shows that the sample consisted of parallelly arranged fibers with a width of 0.5–1.0 μm and a length of 50–100 μm. HRTEM and SAED (Figure 6b–d) images document that the sample was the most defective and feature-rich of the studied amphiboles. CMFs were pervasive, together with a diffuse presence of Moiré fringes, that were a consequence of a slight misorientation of superposed crystal lattices; in the present case they could also be related to a lattice parameter variation due to the presence of lattice defect, i.e., CMF. The surface of many fibers was covered by an amorphous layer. As is the case for all investigated amphiboles, this surface layer, when present, could be identified as a Si-rich amorphous material or as ferrihydrite; additional higher-resolution analytical techniques must be used to distinguish between the two materials without inducing beam-damage artefacts in the studied region that eould lead to erroneous conclusions. Microchemical data of this studied UICC crocidolite (Table 3) show that the only significant departure of the amphibole from an end-member riebeckite composition was the presence of low, but significant amounts of Mg (0.52 apfu) at *M*(1,3); the ferric iron content was also very close to the ideal value (Fe^3+^ = 2.05 apfu). Trace elements included Ti, Cr, Ni, Mn, and Ca.

The FTIR spectrum of the UICC crocidolite in the OH-stretching region (Figure 5) shows three well-resolved peaks at 3650, 3636, and 3618 cm^−1^. This pattern is almost identical to that of a well-characterized sample from Madagascar published recently in Reference [39] for the synthetic end-member riebeckite [51], and the UICC amosite (Figure 5). A broad absorption extending from 3710 to 3680 cm^−1^ could be assigned to small amounts of alkali cations at the A-site [51]. From the relative intensities of the component bands fitted to the spectrum of Figure 5, neglecting the broad absorption peak at 3690 cm^−1^ provided a Fe^2+^ content at *M*(1,3) = 2.39 apfu. Intriguingly, the high-temperature oxidation of crocidolite has been the object of a large number of studies during the 1960–1970s due to the increasing technological relevance of this material, whose widespread use was abandoned only once its carcinogenic effect was definitively recognized. A detailed study has been provided recently in Reference [39], which showed that this process is far from being straightforward and may have significant implications for the surface availability of iron species.

#### 4.1.4. Tremolite Asbestos from Val d’Ala

Tremolitic amphiboles belong to the calcium-amphibole subgroup, defined by ^B^(Ca + ΣM^2+^)/ΣB ≥ 0.75, ^B^Ca/ΣB ≥ ^B^ΣM^2+^/ΣB [42]. Ideal tremolite has the formula ^A^(□)^B^(Ca)_2_^C^(Mg)_5_^T^(Si)_8_O_22_^W^(OH)_2_, with a nominal solid-solution towards the end-member ferro-actinolite ^A^(□)^B^(Ca)_2_^C^(Fe^2+^)5^T^(Si)_8_O_22_^W^(OH)_2_; all compositions along this join have *C*2/*m* symmetry [42]. Intermediate ^C^(Mg,Fe^2+^) compositions are termed “actinolite”.

Actinolites are essentially metamorphic minerals occurring in both contact and regionally metamorphosed rocks, whereby they are particularly common in the latter environment [7]. Actinolites are characteristic of greenschist-facies metabasites (ophiolitic sequences), whereas tremolite occurs in metacarbonates and metamorphosed ultrabasic rocks. Actinolites occur typically with extremely fibrous shapes, and their growth is in many cases connected to rock deformation, which is accompanied by fluid-rock interactions during the geological history of the parent rocks [66,67].

Mg-rich members are usually more fibrous than the Fe-rich ones, a feature that has been observed during synthesis of similar systems, such as along the richterite-ferro-richterite join [64], and they possess appreciable tensile strength [7]. Tremolite, as well as the other regulated amphibole of the calcium subgroup, i.e., actinolite, has not been commercially exploited, but it occurs in the environment as contaminants in chrysotile asbestos, vermiculite, and talc. In the absence of a UICC reference material, the sample described here is from a well-known occurrence of fibrous tremolite, namely from the serpentinites associated with the ultrabasic Massif of Lanzo outcropping at Ala di Stura (Val d’Ala), Piedmont, Italy [37,40].

**Figure 7.**
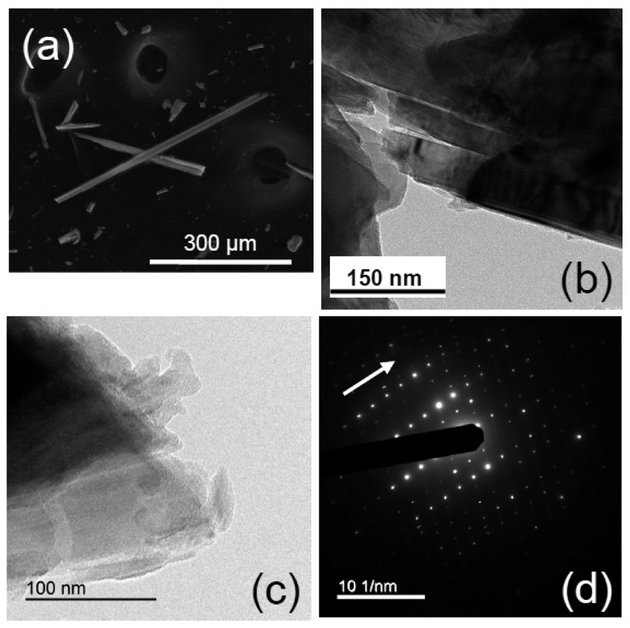
(**a**) Representative FEG-ESEM images of the typical appearance of tremolite from Val d’Ala, Turin (Italy) (Table 1). (**b**) TEM medium high-resolution images displaying cleavage fragments that were detaching from the main fibre, with disoriented Moiré fringes, (**c**) HRTEM image showing highly irregular edge features. (**d**) Representative SAED pattern oriented parallel to the c* axis.

Previous XRPD studies showed the studied sample to consist of tremolite with minor amounts of antigorite, clinochlore, hematite, and quartz [34]. Refined unit-cell data (Table 2) match those measured in similar minerals [28]. In contrast to amosite, the measured *β* angle was close to 105°, in agreement with the presence of a large divalent cation (Ca) at the B-site (Table 2). The tremolite from Val d’Ala consisted of asbestiform, bladed and acicular crystals, showing a tendency to split apart in fibers (mostly at the micro-scale) and/or cleavage fragments (down to the nanoscale) (Figure 7a). TEM images of the Val d’Ala tremolite (e.g., Figure 7b) document the separation of elongated fiber-like particles and/or cleavage fragments derived from the main fibers or from elongated bladed particles. The diffuse presence of Moiré fringes suggests a planar ((110) or (200)) rearrangement of crystal planes (Figure 7b). The apex of the fibers shows evidence of dissolution and re-precipitation processes, with subsequent accumulation of probably Si-rich amorphous or re-crystallized material characterized by an irregular shape and multiple crystallographic orientations (Figure 7c). The SAED (Figure 7d) and HRTEM investigations did not provide evidence of structural defects. Microchemical data and the derived crystal-chemical formula of this sample from Val d’Ala, provided in References [34,37], show that the amphibole was close to the tremolite end member, as it had a very low Fe^2+^ content (0.22 apfu) and only negligible amounts of Fe^3+^ (0.08 apfu). Trace elements included Ti, Cr, Ni, Mn, and alkalis (Table 3).

The FTIR spectrum of the studied tremolite in the OH-stretching region (Figure 5) shows three well-resolved peaks at 3673, 3656, and 3643 cm^−1^, which could be assigned to the different Mg/Fe^2+^ configurations at *M*(1,3) [26,64]. No peaks due to the OH-containing impurities, such as antigorite and clinochlore, are observed in the spectrum, in agreement with their very low abundance determined by XRPD (see above). From the relative intensities of the component bands fitted to the spectrum of Figure 5, a Fe^2+^ content at *M*(1,3) of 0.35 apfu was derived, in excellent agreement with the site occupancies refined via the Rietveld method in Reference [40].

#### 4.1.5. Actinolite Asbestos from Valle Aurina

The sample described here was from a well-known occurrence of fibrous tremolite in Valle Aurina (northern Italy). It has been fully described in Reference [41].

According to the high-resolution synchrotron radiation XRPD analysis, the studied sample consisted of pure amphibole within the detection limit of the method. The refined unit-cell data (Table 2) are in agreement with previous data for similar amphiboles [28]. FEG-FESEM images [41] show that the sample consists of both fibrous and acicular crystals, and the HRTEM images and indexed electron diffraction spots provide evidence for a high degree of crystallinity of the amphibole and absence of dislocations [41]. Published EMPA data for actinolite from Valln Aurina (Table 3) show that the amphibole had an intermediate Fe^2+^ contend (0.f9 apfu), with minor Fe^3+^ (0.11 apfu) and traces of K, Na, Nit, and Cr.

The FTIR spectrum of this actinolite in the OH-stretching region (Figure 5) shows three well-resolved peaks at 3673, 3658, and 3643 cm^−1^, which could be assigned to the Mg/Fe^2+^ configurations at *M*(1,3), similarly to what was (discussed above for tremolite from Val d’Ala. The stronger intensity of the 3658 and 3643 cm^−1^ penks in the OH-spectrum of actinolite compared to that of tremolite is in agreement with its higher Fe^2+^ content (Table 3). From the relative intensities of the component bands fitted to the spectrum of Figure 5, a Fe^2+^ content at *M*(1,3) of 0.91 apfu was derived, in excellent agreement with the site occupancies refined via the Rietveld method in Reference [41].

### 4.2. FTIR Spectra in the Low-Wavenumber Region

As stated above, the interpretation of the vibrational spectra of amphiboles in the region <1200 cm^−1^ and, in particular, at <650 cm^−1^, was extremely challenging due to the complexity of the spectra, and because in this range, tetrahedral modes overlap with octahedral (M-O) modes and OH librations [26]. In addition, the internal vibrations of the T_4_O_11_ double chain involve different types of T-O bonds, as a result of the occurrence of both bridging (O_b_: O(5), O(6), O(7)) and non-bridging (O_nb_: O(1), O(2)) oxygens (Figure 1b). References [68–70] presented a factor group analysis of the vibrations that are IR-active for the T_4_O_11_ unit of the amphiboles, but assignment of the many bands in this region is still done on an empirical basis [71,72]. Despite these difficulties, vibrational spectroscopy in the lattice region is extremely useful and widely applied for asbestos identification, particularly with micro-Raman spectroscopy [73–76]. The success of this method is essentially due to the fact that the Raman spectrum in the low-frequency region provides a reliable fingerprint for regulated asbestos species; the high-resolving power of Raman (beam dimension < 1 μm^2^) also makes this technique suitable for biomedical/toxicological studies [77,78]. A comprehensive summary of Raman data for amphiboles in the region of framework phonon modes has been provided recently in Reference [26]. FTIR data for the <1200 cm^−1^ region are relatively scarce, however, and very few applications to asbestos minerals exist [79], although this technique might be extremely useful in environmental studies due to the ease of sample preparation and data acquisition/interpretation.

The few systematic FTIR studies performed on relatively well-constrained solid-solution series clearly show that the lattice-mode IR spectra are extremely sensitive to the chemical variations at the different structural sites. According to References [71,72], the MIR range of amphiboles may be conveniently divided into three regions: (1) The 1200–800 cm^−1^ region, where seven to eight very intense to medium intense bands due to lattice Si-O-Si and O-Si-O antisymmetric stretching vibrations are observed. In particular, the highest wavenumber peaks in this range can be assigned to T(1)-O(1), which is a very short bond in the amphibole structure [80]. (2) The 800–600 cm^−1^ range, where four to six medium weak to very weak peaks resulting from Si-O-Si symmetric stretching or chain deformation modes occur; in this range two OH librational modes at around 700 and 690–640 cm^−1^ have been identified in ^T^Al-free amphiboles, via hydrothermal treatment with deuterium [45]. The most intense peak in the Raman spectrum of amphiboles occurs in this region, and it has been shown that the wavenumber value of this sharp peak provides a reliable identification of the species [20,77]. (3) At wavenumbers <650 cm^−1^, there is a mixture of T-O and M-O bending modes and OH librations [81]. For alkali amphiboles of the glaucophane-riebeckite series, Ishida et al. [71] reported linear shifts for most bands in this region, consistent with the strong influence of the chemical composition of the amphibole on the peak positions.

**Figure 8.**
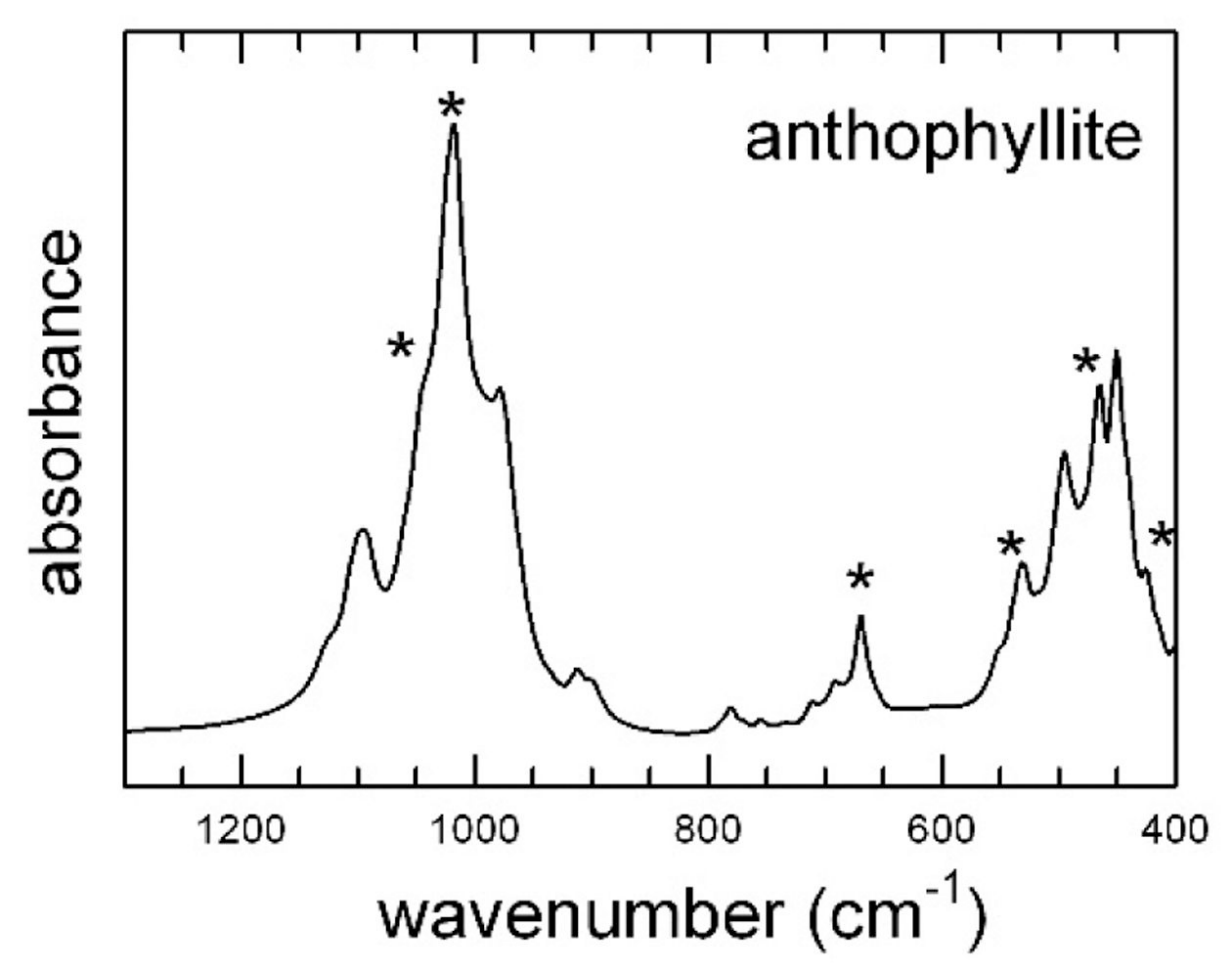
FTIR spectrum of UICC anthophyllite in the low-frequency (1300–400 cm^−1^) region. The asterisks indicate the main bands of talc [45].

**Figure 9.**
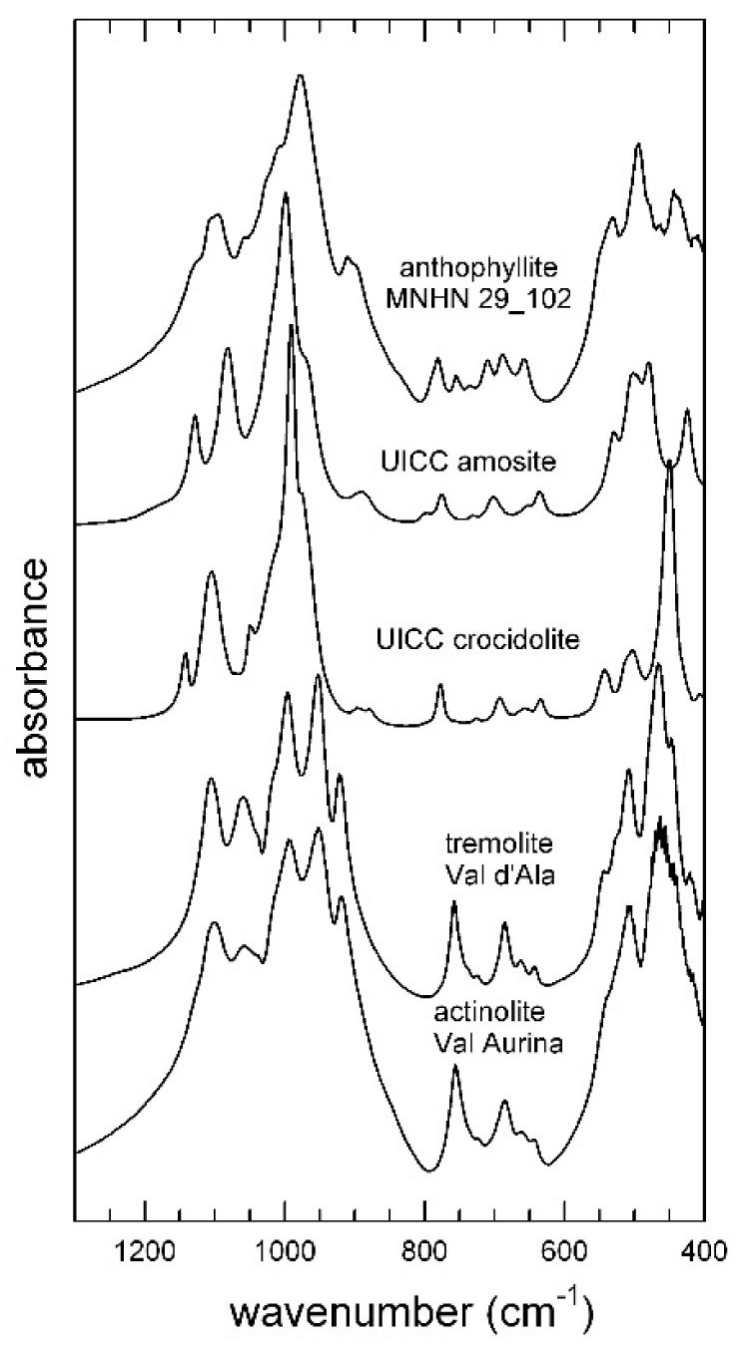
Stacked FTIR spectra in the low-frequency (1300–400 cm^−1^) region for the amphiboles investigated in this study (see Table 1).

The spectra collected in the 1300–400 cm^−1^ range for the studied amphiboles are given in Figures 8 and 9. In particular, Figure 8 shows the spectrum collected for UICC anthophyllite, a sample that, as discussed above, contains significant impurities of hydrous silicates (talc and chlorite); the sharp peaks at 1041, 1091, 669, 531, 465, and 424 cm^−1^, indicated with an asterisk in Figure 8, could be assigned to talc [45]. In Figure 9, the spectra of all other samples are displayed; relevant data and tentative band assignment from the literature are given in Table 4. Inspection of Figure 9 reveals that, in region 1, the patterns of tremolite and actinolite were almost identical, with the only chemical difference between these samples being the Mg-Fe^2+^ substitution at the C-sites. Five intense bands plus two shoulders were observed (Table 4). In the same region, however, the spectra of UICC anthophyllite, amosite, and crocidolite were significantly different and show, as a peculiar characteristic, a major and sharp peak plus several medium intense bands and shoulders. Inspection of the literature data unveiled that the spectrum of anthophyllite MNHN29_102 is almost identical to those of anthophyllites published in Reference [46], whereas the spectrum of UICC crocidolite is almost identical to that of riebeckites published in References [71,82], suggesting that the IR patterns in this region are indeed characteristic of the species.

Following the work of Reference [46], the positions of the medium-strong peaks in region 2 are also characteristic of the amphibole species, being sensitive of both the A- and B-site occupants. In the present case, the A-site was empty for all samples, thus the observed differences were due to the B-site cation only: for Na (crocidolite), there was a single and relatively sharp peak at 778 cm^−1^; for Mg (anthophyllite), a very weak single band at 780 cm^−1^; for Fe^2+^ (amosite), a doublet at 797–775 cm^−1^; and for Ca (tremolite-actinolite), a medium intense peak at 757 cm^−1^. The OH librations, identified through deuteration experiments, occurred at 709 and 688 cm^−1^ for anthophyllite [46] and at 685 and 642 cm^−1^ (Table 4) for tremolite [72].

Significant differences were finally observed in region 3, and these are again related to the amphibole type: the Fe-Mg amphiboles anthophyllite and amosite; showed a medium-intensity peak around 530 cm^−1^ followed by an intense doublet around 480–500 cm^−1^. In the Na-amphibole crocidolite at the same wavenumbers, there are two weak peaks, followed by a sharp and intense absorption at 450 cm^−1^. The Ca-amphiboles tremolite and actinolite show two/three peaks with medium intensity at 600–500 cm^−1^ followed by an intense doublet at 445–465 cm^−1^.

## 5. Conclusions

This work is intended to fill a gap in vibrational spectroscopy of asbestos amphiboles, providing FTIR data for a suite of well-characterized regulated species, whose IR data are either scattered or not present in the literature. “The data presented here can be used ns reference spectra for environmental/toxicological studies where vibrational spectroscopy is used as an analytical tool. Our study shows that, based on a vast body of literature, mostly summarized in References [21,26], the FTIR spectra in the OH-stretching region provide a relatively easy and rapid identification of the amphibole asbestos minerals; this task is eased by the fact that all asbestos amphiboles have a vacant A-site, a feature that has a strong effect on the IR spectra [21,30] and references therein. Considering that the exact wavenumbers of the OH-stretching bands are also sensitive, albeit weakly, to the next-nearest neighbors, i.e., the *M*(4) and *M*(2) cations [8,51,57], the calcic amphiboles tremolite–actinolite can be easily distinguished from the ferromagnesian amphiboles of the anthophyllite-grunerite type. For simple binary ^*M*(1,3)^Mg-^*M*(1,3)^Fe^2+^ so1id-solutions, as is typically the case for asbestos amphiboles, the relative amount of these cations in the OH-bonded octahedra can be calculated with a high degree of confidence [26], and, provided that the Al and Fe_tot_ amounts are known from EMPA, the complete cation distribution at all octahedral sites can be derived.

The present work also shows that, although data from species spanning the complete chemical diversity of this large mineral supergroup are still lacking, the spectra in the mid-IR lattice modes region can be effective for rapid identification of the asbestos type.

## Figures and Tables

**Table 1.** Studied samples.

Amphibole Species	Provenance	Reference
Anthophyllite	UICC standard Anthophyllite from Paakkila, NB #4173-111-5	[34,35]
Anthophyllite	AMNH34856, Talcville, New York State (USA)	[36]
Anthophyllite	MNHN29_102	Della Ventura, unpublished
Amosite	UICC standard Amosite South African NB #4173-111-4	[34,35]
Crocidolite	UICC standard Crocidolite South African NB #4173-111-3	[34,37,38]
Crocidolite	Madagascar	[39]
Tremolite	Val d′Ala, Turin (Italy)	[34,40]
Actinolite	Valle Aurina (Bolzano, Italy)	[41]

**Table 2.** XRD data for the studied reference amphiboles.

Sample	*a* (Å)	*b*(Å)	*c*(Å)	*β*(°)	Space Group	Ref.
Anthophyllite UICC	18.5770(8)	18.0353(22)	5.27285(9)	90	*Pnma*	[35]
Amosite UICC	9.5484(2)	18.3395(4)	5.3346(1)	101.825(2)	C2/*m*	[35]
Crocidolite UICC	9.73238(7)	18.0414(1)	5.32794(4)	103.515(1)	C2/*m*	[34]
Tremolite Ala	9.8424(1)	18.0712(2)	5.28354(7)	104.680(1)	C2/*m*	[40]
Actinolite Aurina	9.84896(4)	18.07748(8)	5.28860(2)	104.817(2)	C2/*m*	[41]

**Table 3.** Microchemical data for the studied reference amphiboles.

Sample	Anthophyllite UICC	Amosite UICC	Crocidolite UICC	Tremolite	Actinolite
reference	[35]	[35]	[34]	[40]	[41]
SiO_2_	57.3(4)	49.8(2)	51.7(3)	57.8(1)	54.83(5)
TiO_2_	0.02(2)	0.04(2)	0.03(3)	0.02(2)	0.04(9)
A1_2_O_3_	0.11(5)	0.04(2)	0.12(4)	0.11(7)	2.70(6)
FeO	9.9(7)	37.8(4)	17.3(9)*	2.4(4)*	6.76(5)
Fe_2_O_3_	0.0	3.3(4)	18.7(9)*	0.3(4)*	1.01(5)
Cr_2_O_3_	0.04(3)	0.01(1)	0.01(1)	0.02(2)	0.08(9)
NiO	0.04(4)	0.02(4)	0.01(2)	0.08(5)	0.18(2)
MnO	0.4(1)	0.42(6)	0.04(2)	0.15(5)	0.40(7)
MgO	28.5(2)	6.23(9)	2.3(4)	22.8(2)	18.21(3)
CaO	0.27(7)	0.09(1)	0.22(7)	12.9(1)	12.77(6)
Na_2_O	0.02(1)	0.03(2)	6.7(2)	0.23(6)	0.48(6)
K_2_O	0.02(1)	0.02(1)	0.05(4)	0.05(2)	0.13(3)
H_2_O	2.30	1.94	2.18	2.02	2.47
Total	98.92	99.74	99.36	98.88	100.06

* Partition determined from Mössbauer spectroscopy [44].

**Table 4.** Peak positions (cm^−1^) and band assignment (according to 71) for the studied amphiboles in the low-wavenumber (<1200 cm^−1^) range.

Anthophyllite 29_102	Amosite UICC	Crocidolite UICC	Tremolite	Actinolite	Assignment
1126	1128	1142	1105	1102	**Region 1** Si-O-Si, O-Si-O and Si-O stretching modes
1106	1082	1105	1059	1058	
1095	1020	1048	1038	1038	
1058	997	1017	1015	1012	
1026	969	990	995	993	
1008	888	976	953	952	
978		896	921	919	
910		878			
898					
781	797	778	757	756	**Region 2** chain deformations and OH libration modes
755	775	726	738	736	
736	729	693	724	724	
709	700	655	685	684	
688	654	634	661	661	
657	635		642	642	
530	528	543	543	540	**Region 3** M-O modes
495	499	506	526	508	
480	479	450	508	463	
463	424		465	446	
440			445		
